# Effects of Melatonin on Morphological Characteristics, Mineral Nutrition, Nitrogen Metabolism, and Energy Status in Alfalfa Under High-Nitrate Stress

**DOI:** 10.3389/fpls.2021.694179

**Published:** 2021-06-29

**Authors:** Zhao Chen, Xinlong Cao, Junpeng Niu

**Affiliations:** College of Grassland Agriculture, Northwest A&F University, Yangling, China

**Keywords:** melatonin, mineral nutrition, nitrogen metabolism, energy status, high-nitrate stress, alfalfa

## Abstract

Melatonin is an indoleamine small molecular substance that has been shown to play an important role in the growth, development, and stress response of plants. The effects of melatonin on the morphological characteristics, mineral nutrition, nitrogen metabolism, and energy status in alfalfa (*Medicago sativa* L.) under high-nitrate stress were studied. The alfalfa plants were treated with water (CK), 200 mmol L^−1^ nitrates (HN), or 200 mmol L^−1^ nitrates + 0.1 mmol L^−1^ melatonin (HN+MT), and then were sampled for measurements on days 0 and 10, respectively. The results showed that the HN treatment resulted in a decrease in the morphological characteristics (such as shoot height, leaf length, leaf width, leaf area, and biomass), phosphorus, soluble protein (SP), nitrogen-related enzymes activities and gene relative expression, adenosine triphosphate (ATP), and energy charge (EC). It also caused an increase in nitrogen, sodium, potassium, calcium, nitrate-nitrogen (${\text{NO}}_{3}^{-}$-N), ammonium-nitrogen (${\text{NH}}_{4}^{+}$-N), adenosine diphosphate (ADP), and adenosine monophosphate (AMP). However, these parameters were conversely changed in the HN+MT treatment. Besides, these parameters were closely related to each other, and were divided into two principal components. It reveals that melatonin plays an important role in modulating the morphology, mineral nutrition, nitrogen metabolism and energy status, thereby alleviating the adverse effects of high-nitrate stress and improving the growth of alfalfa.

## Introduction

Melatonin (N-acetyl-5-methoxytryptamine) is a tryptophan-derived low molecular weight indole amine that is widespread in bacteria, algae, animals, and higher plants (Back et al., 2016; Zhang J. et al., 2019). Melatonin is also a universal signaling molecule in mammals and plant species (Zhang Q. et al., 2019; Zhao et al., 2021). As an important free radical scavenger and antioxidant, melatonin is involved in various physiological processes, and it modulates plant tolerance or resistance to abiotic stresses, including salinity, drought, heat, cold, water, heavy metal, nutritional deficiency, osmotic and ionic stress, ultraviolet radiation and chilling injury (Li et al., 2012; Tan et al., 2012; Shi and Chan, 2014; Shi et al., 2015; Antoniou et al., 2017; Gong et al., 2017; Zhang R. et al., 2017; Zhao et al., 2017; Kobylińska et al., 2018; Zhang Q. et al., 2019; Bose and Howlader, 2020). Hence, melatonin plays an essential role in improving plant growth, development and protecting plants from adverse conditions.

Alfalfa (*Medicago sativa* L.) is a widely cultivated perennial legume species in the world (Wolabu et al., 2020). As the most productive and highest quality forage crop, alfalfa can be used for grazing, silage, hay, and even human fresh food and health products (Tang et al., 2013; Jaime et al., 2019; Lin et al., 2020). Nitrogen is a component of amino acids, nucleic acids, proteins, nucleoproteins, chlorophyll, enzymes, hormones and secondary metabolites, as well as the basis of key genetic materials (Wen et al., 2020). Hence, the efficiency and availability of nitrogen have decisive influences on the growth, development, and metabolisms of plants (Hachiya and Sakakibara, 2017). For most plants, nitrate-nitrogen (${\text{NO}}_{3}^{-}$-N) and ammonium-nitrogen (${\text{NH}}_{4}^{+}$-N) are major sources of inorganic nitrogen (Zhu et al., 2020). Although alfalfa can fix nitrogen through nodules, the amount of nitrogen fixation by nodules is 50 to 60% of its lifetime nitrogen demand. That is, root nodule nitrogen fixation cannot meet the normal growth needs of alfalfa. Furthermore, the soil of most alfalfa fields is low in ${\text{NO}}_{3}^{-}$-N and organic matter, and these areas suffer from salinization, drought, and low temperatures. Therefore, rational application of nitrogen fertilizer is a guarantee for good growth, high quality and high yield of alfalfa, especially in areas with poor soils and harsh climates (Zeng et al., 2006; Liu et al., 2015; Yu and Ma, 2015; Hao et al., 2017).

${\text{NO}}_{3}^{-}$-N is the most prevalent in soil for the growth and morphological construction of most plants (Oldroyd and Leyser, 2020; Wen et al., 2020). However, in the alfalfa-growing areas with a dry climate and alkaline soil, soil nitrification is serious but denitrification is weak, and excess ${\text{NO}}_{3}^{-}$-N is easy to accumulate in the soil (Ju et al., 2011). Besides, the excessive and unreasonable application of nitrogen fertilizer also affects the growth and yield of alfalfa in recent years (Hao et al., 2015, 2017). It has demonstrated that excessive ${\text{NO}}_{3}^{-}$-N reduces photosynthesis and enzyme activities, and increases ionic toxicity, osmotic stress and reactive oxygen species (ROS), further affecting crop quality and yields (Yang et al., 2010; Zhang R. et al., 2017). Nitrate absorption and transformation are mainly regulated by the nitrogen metabolism pathway in alfalfa, which is mainly modulated by nitrate reductase (NR), glutamine synthetase (GS), glutamate synthase (GOGAT), and glutamate dehydrogenase (GDH) (Zhang R. et al., 2017; Wen et al., 2020). In addition, plant growth and development are ultimately driven by light energy captured through photosynthesis, and the energy available state plays an important role in the metabolism of plants (De Col et al., 2017). Therefore, nitrogen mechanism and energy status are of great significance to the growth and yield of alfalfa under high ${\text{NO}}_{3}^{-}$-N.

The roles of melatonin in regulating alfalfa adaptation to high-nitrate stress are seldom studied. In this study, the objective was to evaluate the effects of melatonin on morphological characteristics, mineral nutrition, nitrogen metabolism and energy status in alfalfa under high-nitrate stress. Thus, a comprehensive understanding of the role of exogenous melatonin for improving alfalfa nitrate stress tolerance was provided.

## Materials and Methods

### Plant Growth and Treatments

The seeds of “Sanditi” (*Medicago sativa* L.) were sown in the pots using a 3:1:1 mixture of peat, vermiculite, and pearlite, respectively. Then the pots were placed in a growth chamber at 25°C with a 14-h daily photoperiod (20000 lx) and 10-h dark. At the three-leaf stage, the excess seedlings were removed so that there were nine seedlings in each pot. They were regularly watered. After 15 d, the pots were divided into three groups (six replications per group), namely normal control (CK), high nitrates (HN), and high nitrates + melatonin (HN+MT). The plants were treated with water, 200 mmol L^−1^ nitrates, and 200 mmol L^−1^ nitrates + 0.1 mmol L^−1^ melatonin, respectively. Nitrate and melatonin were used with the optimum concentration selected from the pre-experiment. And nitrate and melatonin were used by irrigation and foliar spraying, respectively. The nitrate was composed of calcium nitrate [Ca(NO_3_)_2_] and potassium nitrate (KNO_3_) averagely. The treatments were applied every other day for a total of three times. Then, three replications of each group were sampled for measurements, which were labeled 0 d. The others continued to grow for 10 d and then were sampled. Shoot height, leaf length, leaf width, leaf area, shoot fresh weight (FW) and dry weight (DW) were measured both on days 0 and 10. The leaf samples were quickly frozen in liquid nitrogen and stored at −80°C for the other parameters.

### Mineral Nutrition

The leaves were sampled and washed three times with distilled water. Then they were fixed at 105°C for 30 min, and dried for 48 h at 65°C. They were ground to powder to pass through a 1-mm sieve. Nitrogen and phosphorus were determined with a Continuous Flow Analyzer (Flowsys; Systea, Anagni, Italy) after wet digestion of H_2_SO_4_-H_2_O_2_. The powder (0.1 g) for sodium, potassium, and calcium was extracted in boiling water with deionized water for 2 h. Then, the concentrations were determined with a Flame Photometer (M410 blue notes, Sherwood, UK).

### ${\text{NO}}_{3}^{-}$-N and ${\text{NH}}_{4}^{+}$-N Concentrations

${\text{NO}}_{3}^{-}$-N and ${\text{NH}}_{4}^{+}$-N concentrations were measured using fresh leaf samples by the methods of Zhang R. et al. (2017). Fresh samples (0.5 g) were ground in 10 mL of water and then held in a boiling water bath for 30 min. The supernatant was diluted to 25 mL. Then, 0.1 mL solution was mixed with 0.4 mL of 5% salicylic-H_2_SO_4_ to react for 20 min. And 9.5 mL of 8% NaOH was added. After cooling to room temperature, absorbance was measured at 410 nm (UV-3000, Mapada, China) for ${\text{NO}}_{3}^{-}$-N. As to ${\text{NH}}_{4}^{+}$-N concentrations, the fresh samples were ground in 10 mL of 10% acetic acid and the volume was made up to 100 mL with distilled water. Then, 2 mL of the solution, 3 mL of ninhydrin hydrate and 0.1 mL of 1% ascorbic acid were mixed and heated in boiling water for 15 min. After being cooled in an ice bath, the mixture was measured at 580 nm (UV-3000, Mapada, China).

### Soluble Protein (SP) and Proline (Pro)

SP concentrations were measured by the method of Li and Zhang (2016). The fresh sample (0.5 g) was ground with 5 mL distilled water and centrifugated at 3,000 g. The supernatant (1 mL) was added to 5 mL of 10% coomassie brilliant blue (containing 90% ethanol and 85% phosphoric acid). After 5 min, the absorbance was recorded at 595 nm (UV-3000, Mapada, China). SP concentration was calculated by the standard curve (y = 5.8243x + 0.0236, R^2^ = 0.9941).

Pro was measured by the method of Li and Zhang (2016). The fresh samples (about 0.4 g) were extracted with 3% sulfosalicylic acid. Then, the extract (2 mL) was added to 2 mL of ice acetic acid and 2 mL of acidic ninhydrin in the boiling water for 30 min. It should be noted that acidic ninhydrin solution was obtained by dissolving 1.25 g ninhydrin in 30 mL glacial acetic acid and 20 mL 6 mol L^−1^ phosphoric acid, stirring and heating (70°C). After 40-min toluene extraction, the upper toluene solution was absorbed to centrifuge for 5 min to determine the absorbance at 520 nm (UV-3000, Mapada, China).

### Enzyme Activities

NR activity was measured by the method of Su et al. (2018). Leaf samples were added to a 10-mL tube with 5 mL of 0.1 mol L^−1^ phosphatic buffer solution (PBS) and 4 mL of 0.2 mol L^−1^ KNO_3_. The sample was vacuumized for 10 min and kept warm at 30°C for 30 min. Then, 1 mL of 30% trichloroacetic acid (TCA) was added and shaken well. The extract (2 mL) was added to 4 mL of 1% sulfanilamide and 4 mL of 0.2% α-naphthylamine. The reaction continued for 30 min at 30°C. Absorbance was read at 520 nm (UV-3000, Mapada, China).

GS activity was assayed by the method of Oaks et al. (1980) with some modifications. The leaf samples were ground in 3 mL of 0.05 mol L^−1^ Tris-HCl (pH 8.0), then were centrifuged at 4°C. The reaction mixture contained a final volume of 1.6 mL with 0.1 mol L^−1^ Tris-HCl and 80 mol L^−1^ hydroxylamine hydrochloride (pH 7.4). Then, 0.7 mL of enzyme extract and 0.7 mL of 40 mmol L^−1^ adenosine triphosphate (ATP) were added to the reaction mixture. After incubation for 30 min at 30°C, 0.2 mol L^−1^ TCA, 0.37 mol L^−1^ FeCl_3_, and 0.6 mol L^−1^ HCl were added to the mixture. After centrifugation at 5,000 g, the absorbance was measured at 540 nm (UV-3000, Mapada, China).

GOGAT activity was assayed by the method of Singh and Srivastava (1986) with some modifications. The method of enzyme extract was the same as that of GS. The reaction mixture contained 0.4 mL of 20 mmol L^−1^ L-glutamine, 0.05 mL of 0.1 mol L^−1^ α-oxoglutarate, 0.1 mL of 10 mmol L^−1^ KCl, 0.2 mL of 3 mmol L^−1^ NADH and 0.5 mL of the enzyme extract in a final volume of 3 mL, made up with 25 mmol L^−1^ Tris-HCl (pH7.6). The decrease in absorbance was recorded for 3 min at 340 nm (UV-3000, Mapada, China).

GDH activity was assayed by the method of Kanamori et al. (1972) with some modifications. The fresh samples were ground in 6 mL of 10 mmol L^−1^ Tris-HCl (pH 7.6, containing 1 mmol L^−1^ MgCl_2_, 1 mmol L^−1^ EDTA, and 1 mmol L^−1^ β-mercaptoethanol). The enzyme extract was obtained after centrifugation at 4°C. The reaction mixture contained 0.3 mL of 0.1 mmol L^−1^ α-oxoglutarate, 0.3 mL of 1 mol L^−1^ NH_4_Cl, 0.2 mL of 3 mmol L^−1^ NADH, and 1 mL of the enzyme extract in a final volume of 3 mL, made up with 0.2 mol L^−1^ Tris-HCl. The decrease in absorbance was recorded for 3 min at 340 nm (UV-3000, Mapada, China).

### Enzyme Genes

Total RNA was extracted from 100 mg leaf samples frozen in liquid nitrogen using an RNA Extraction Kit (TaKaRa Biomedicals, Japan). Quantitative RT-PCR was performed using an ABI StepOne Real-Time PCR System (USA). The primers were designed using DNAMAN software (Table 1). qRT-PCR was carried out in a 20-μL reaction mixture consisting of 10 μL of SYBR Premix Ex Taq II, 0.4 μL of ROX Dye, 0.8 μL of the forward PCR primer, 0.8 μL of the reverse PCR primer, 7 μL of ddH_2_O and 1 μL of diluted template cDNA. The reaction conditions were as follows: 95°C for 30 s, followed by 40 cycles of 95°C for 5 s, and 60°C for 30 s. The relative gene expression levels were calculated using the 2^−ΔΔCt^ formula and standardized with the β*-actin* gene as an internal control.

**Table 1:** Sequences of the primers used for polymerase chain reaction.

**Genes**	**Accession no**.	**Forward primer (5^′^-3^′^)**
*NR*	3g073150-F	AATCGTCGCAAGGAGCAGAATATGG
	3g073150-R	ACAACGCTTGAGCACGGTTCTC
*GS*	8g062840-F	TTCGTCACCAGGTCCATATTGAAGC
	8g062840-R	ACACAGATTGAGCATCCACAGTTAGC
*GOGAT*	1g027020-F	GTGGTCAGTCGCTTGTGGTATGG
	1g027020-R	CTGTGCTTCTTGTTGAGGTCTTGTTG

### Energy Status

ATP, adenosine diphosphate (ADP), adenosine monophosphate (AMP) and energy charge (EC) were measured by the method of Wang et al. (2017). The samples were ground in liquid nitrogen. Then, 6 mL of 0.6 mol L^−1^ perchloric acid was added to the samples. After a 10-min water bath, the homogenate was centrifuged at 8,000 g and 4°C for 15 min. The pH of the supernatant was neutralized to 6.8 using 1 mol L^−1^ KOH. The solution was diluted to 4 mL and passed through a 0.22-μm filter after another incubation step on ice and centrifugation. Then, 20 μL of the filter liquor was used to measure the concentrations of ATP, ADP, and AMP at 254 nm with an UltiMate 3000 HPLC (Thermo Fisher, Waltham, MA, USA) with a C18 column (5 μm; 250 × 4.6 mm; Phenomenex, USA). Mobile phase A (pH 7) consisted of 60 mmol L^−1^ K_2_HPO_4_ and 40 mmol L^−1^ KH_2_PO_4_, whereas mobile phase B was pure acetonitrile. The flow rate was 1 mL min^−1^. ATP, ADP, and AMP concentrations were calculated via standard curves, while the EC was calculated with the formula: EC = (ATP + 0.5 × ADP)/(ATP + ADP + AMP).

### Statistical Analysis

The experimental data were sorted and calculated using Excel 2019 (Microsoft Corp., Redmond, WA, USA), and were subjected to an analysis of variance (ANOVA) using SPSS 20 Statistics (SPSS Inc., Chicago, IL, USA). A significant difference was indicated at *p* < 0.05.

## Results

### Morphological Parameters

**Figure 1:**
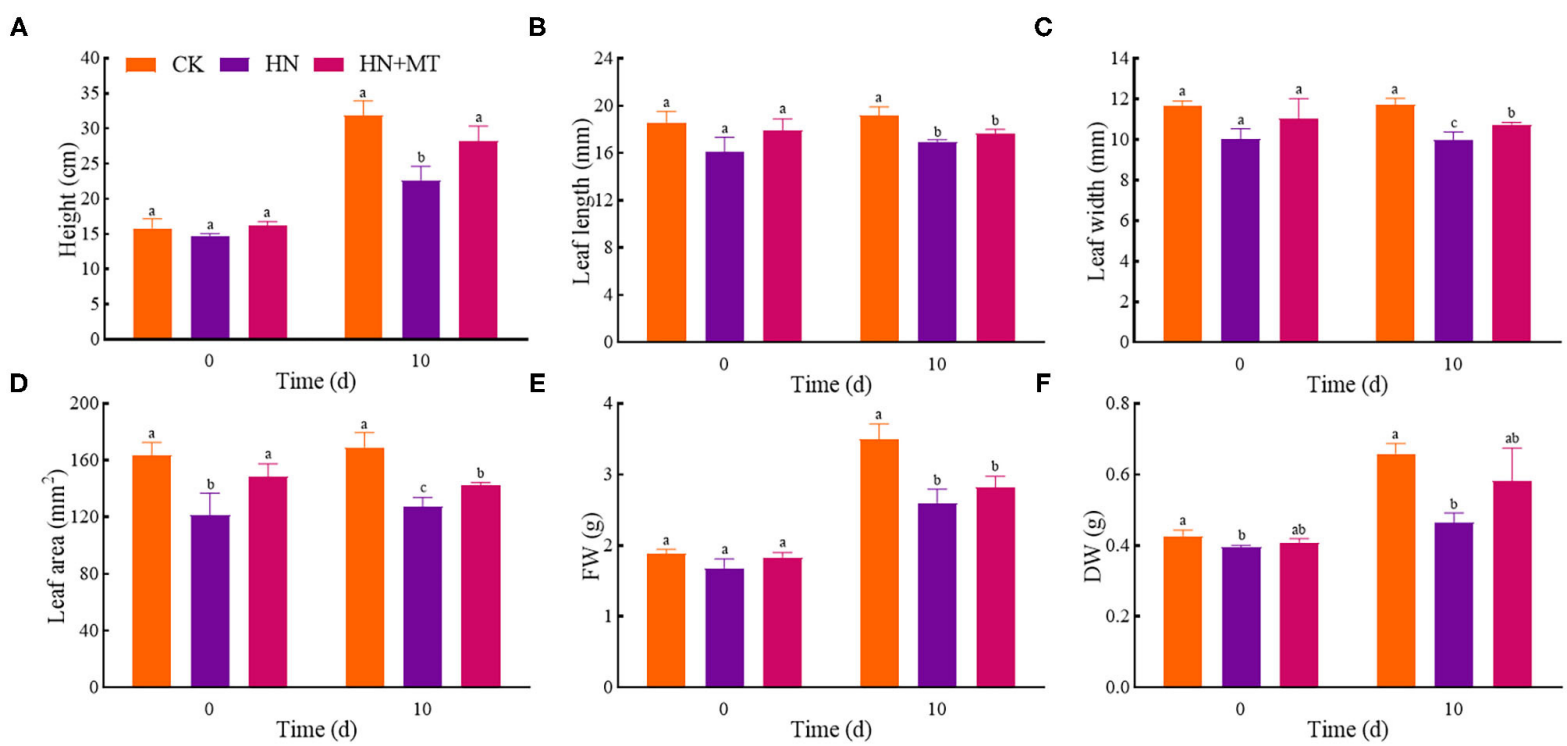
Changes of shoot height **(A)**, leaf length **(B)**, leaf width **(C)**, leaf area **(D)**, FW **(E)**, and DW **(F)**. The groups on the same day followed with the same letter are not significantly different at *p* < 0.05. Bars indicate the SDs of the means.

On day 0, the height, leaf length, leaf width, leaf area, FW and DW were the lowest, and there were significant differences in the leaf area and DW between the HN treatment and the CK (Figure 1). The HN+MT treatment increased these six parameters compared with the HN treatment, whereas they were still lower than the CK with no differences. On day 10, the HN treatment significantly decreased these six parameters compared with the CK. Whereas, the HN+MT treatment increased them, even dramatic differences were observed in the height, leaf width and leaf area.

### Mineral Nutrition

**Figure 2:**
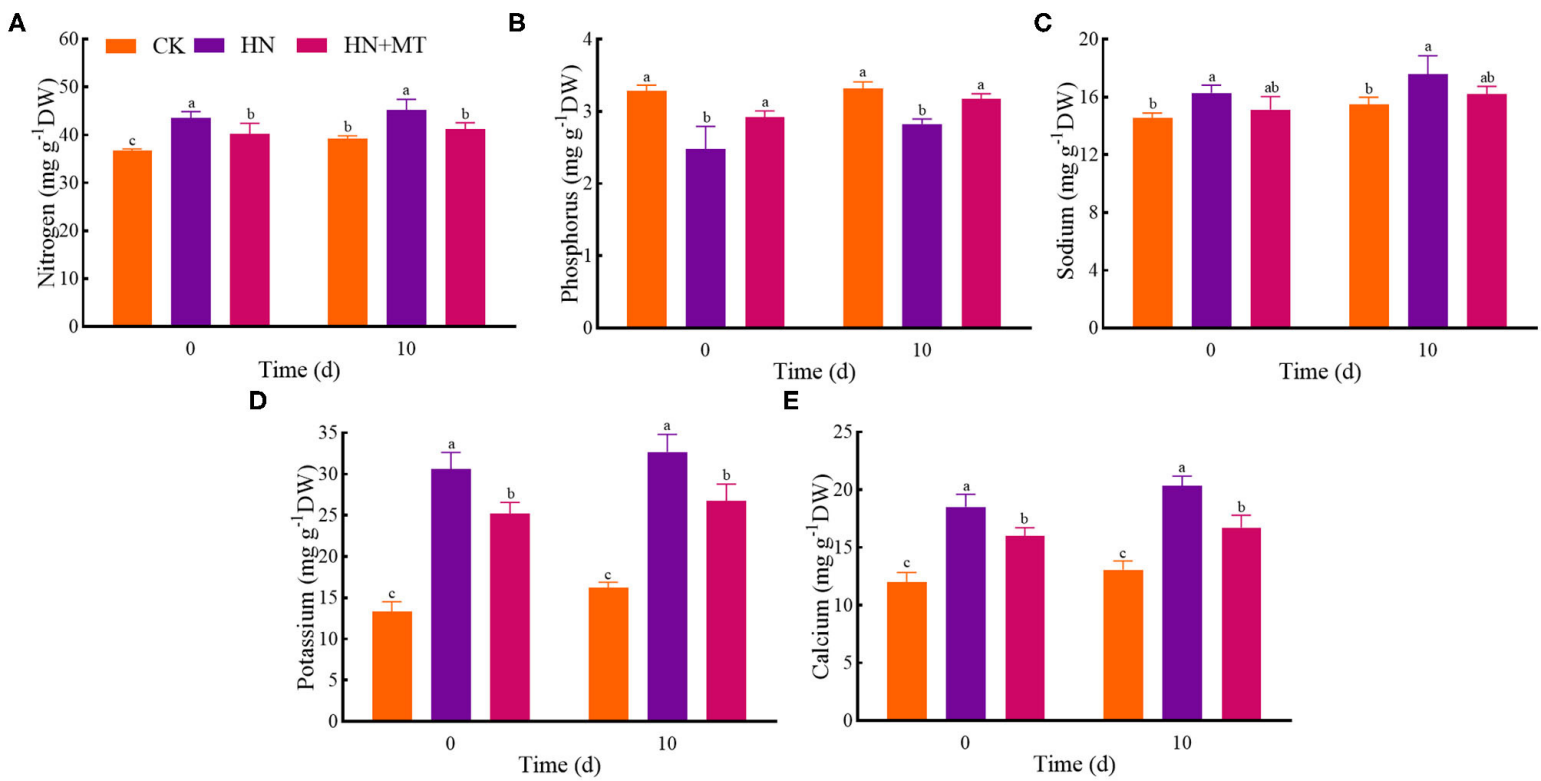
Changes of nitrogen **(A)**, phosphorus **(B)**, sodium **(C)**, potassium **(D)**, and calcium **(E)**. The groups on the same day followed with the same letter are not significantly different at *p* < 0.05. Bars indicate the SDs of the means.

In the HN treatment, nitrogen, sodium, potassium, and calcium had the highest values whereas phosphorus had the lowest value both on days 0 and 10 (Figure 2). Compared with the HN treatment, the HN+MT treatment almost dramatically decreased nitrogen, sodium, potassium, and calcium concentrations but increased phosphorus concentrations. It indicates that melatonin inhibits nitrogen, sodium, potassium and calcium but increases phosphorus in alfalfa under high-nitrate stress.

### Nitrogen Types

**Figure 3:**
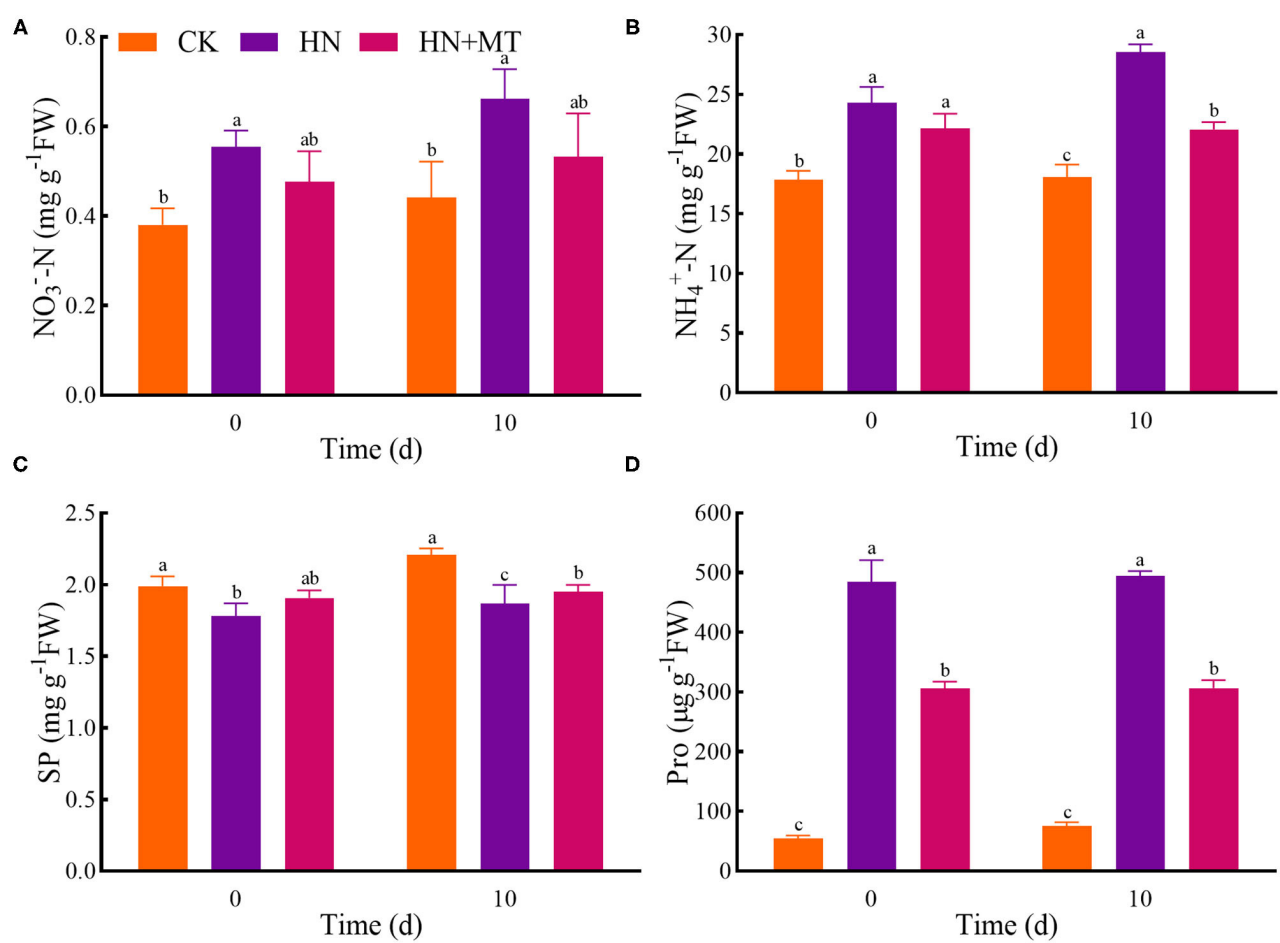
Changes of ${\text{NO}}_{3}^{-}$-N **(A)**, ${\text{NH}}_{4}^{+}$-N **(B)**, SP **(C)**, and Pro **(D)**. The groups on the same day followed with the same letter are not significantly different at *p* < 0.05. Bars indicate the SDs of the means.

Compared with the CK, the HN treatment significantly increased the concentrations of ${\text{NO}}_{3}^{-}$-N, ${\text{NH}}_{4}^{+}$-N, and Pro, while it distinctly restricted the SP concentrations both on days 0 and 10 (Figure 3). The HN+MT treatment showed opposite changes in these four parameters.

### Nitrogen Metabolism Enzymes

**Figure 4:**
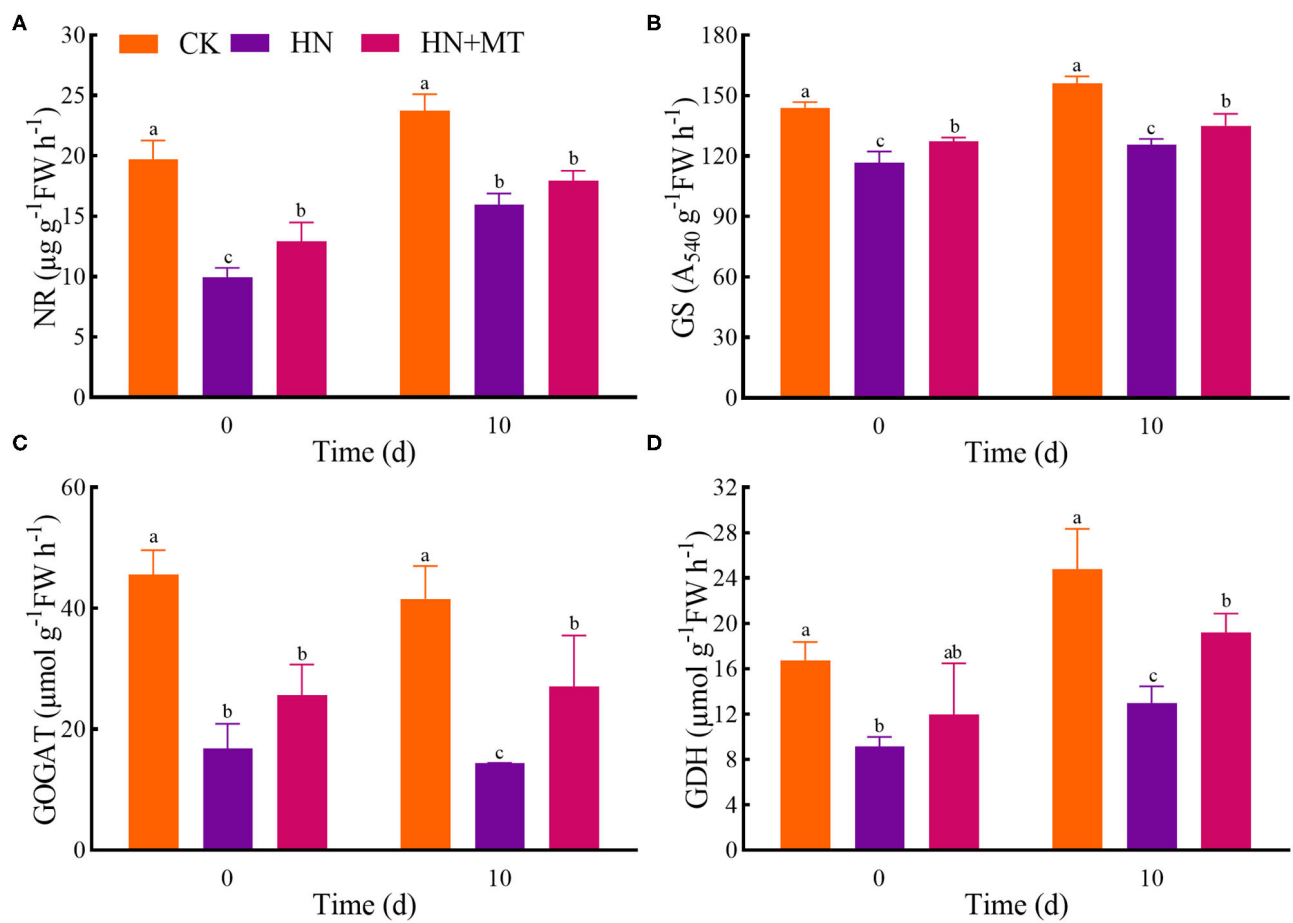
Changes of NR **(A)**, GS **(B)**, GOGAT **(C)**, and GDH **(D)**. The groups on the same day followed with the same letter are not significantly different at *p* < 0.05. Bars indicate the SDs of the means.

The HN treatment significantly inhibited the activities of NR, GS, GOGAT, and GDH, while the HN+MT treatment almost dramatically improved these activities (Figure 4). However, the activities in the HN+MT treatment still were significantly lower than those in the CK.

### Gene Levels of Nitrogen Metabolism Enzymes

**Figure 5:**
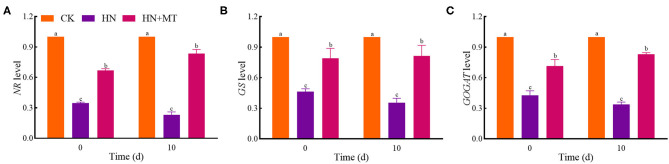
Changes of *NR* **(A)**, *GS* **(B)**, and *GOGAT* **(C)** levels. The groups on the same day followed with the same letter are not significantly different at *p* < 0.05. Bars indicate the SDs of the means.

The expression of genes changed in a way similar to that of enzymes' activities both on days 0 and 10 (Figure 5). The relative expression of *NR, GS*, and *GOGAT* in the HN and HN+MT treatments was lower than those in the CK. The HN treatment significantly down-regulated the genes' expression, whereas the HN+MT treatment distinctly up-regulated the genes' expression.

### Energy Status

**Figure 6:**
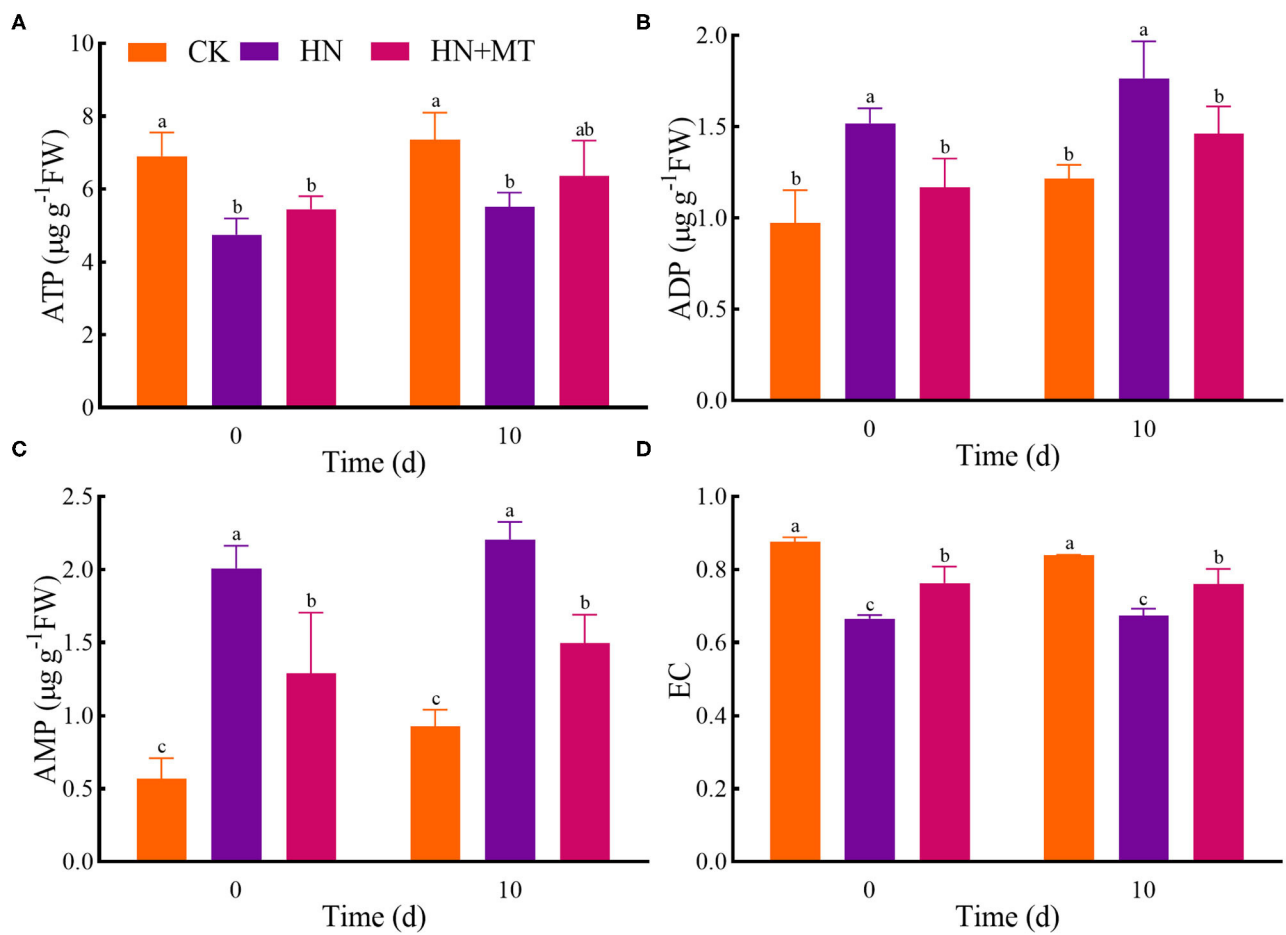
Changes of ATP **(A)**, ADP **(B)**, AMP **(C)**, and EC **(D)**. The groups on the same day followed with the same letter are not significantly different at *p* < 0.05. Bars indicate the SDs of the means.

In the HN treatment, ATP and EC had the lowest value, while ADP and AMP had the highest value both on days 0 and 10 (Figure 6). Conversely, the HN+MT treatment increased ATP and EC whereas decreased ADP and AMP.

### Correlation Analysis and Principal Component Analysis

**Figure 7:**
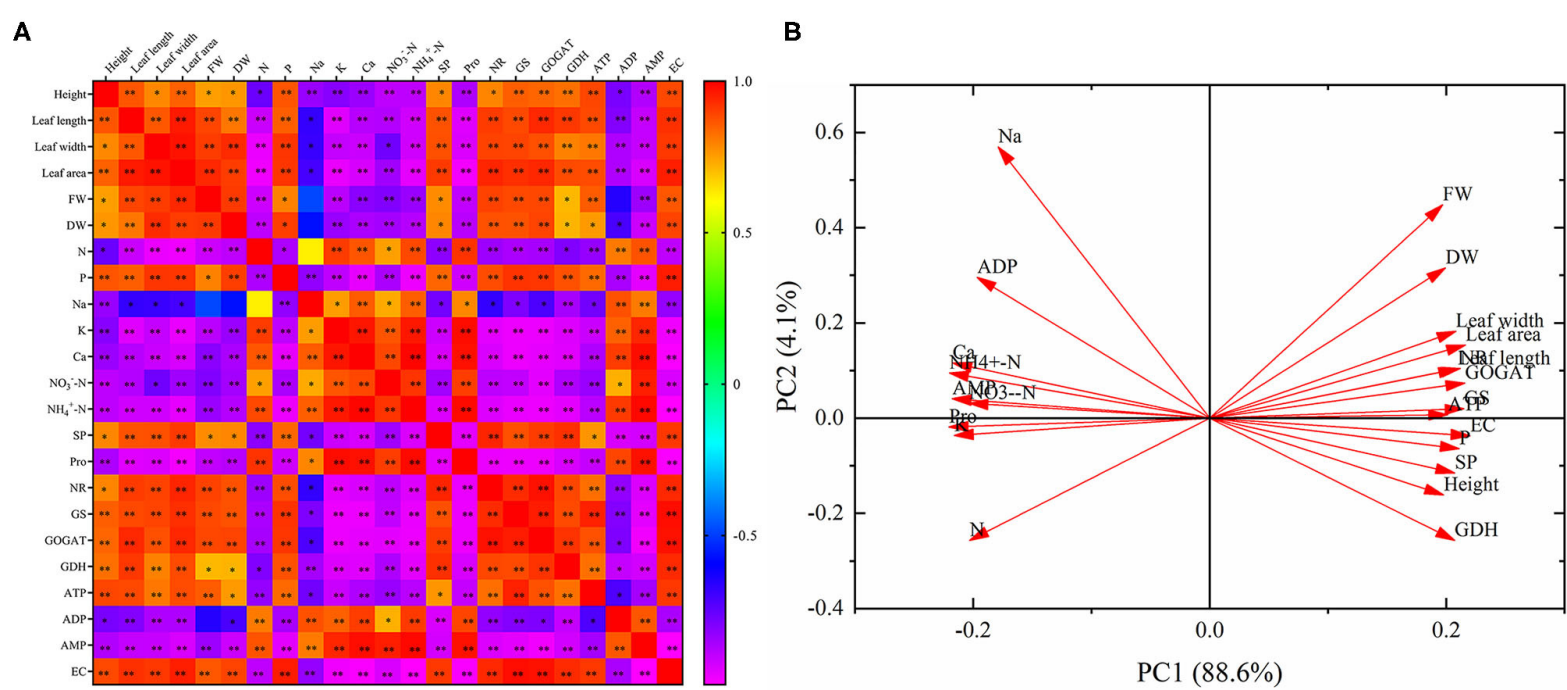
Correlation analysis **(A)** and principal component analysis **(B)**.

Correlation analysis indicates that there was a positive relationship with each other in the height, leaf length, leaf width, leaf area, FW, DW, phosphorus, SP, NR, GS, GOGAT, GDH, ATP, and EC (Figure 7A). And these parameters were negatively correlated with nitrogen, sodium, potassium, calcium, ${\text{NO}}_{3}^{-}$-N, ${\text{NH}}_{4}^{+}$-N, Pro, ADP and AMP, respectively. Nitrogen, sodium, potassium, calcium, ${\text{NO}}_{3}^{-}$-N, ${\text{NH}}_{4}^{+}$-N, Pro, ADP, and AMP were positively correlated with each other. What's more, almost all of the relationships were significant, except for the relationships between FW and sodium, FW and ADP, DW and sodium, nitrogen and sodium.

Principal component analysis reveals that the 23 parameters were divided into PC 1 (88.6%) and PC 2 (4.1%). And PC 1 and PC 2 explained 92.7% of the differences in the 23 indicators. In addition, these 23 parameters were divided into two categories. Nitrogen, sodium, potassium, calcium, ${\text{NO}}_{3}^{-}$-N, ${\text{NH}}_{4}^{+}$-N, Pro, ADP, and AMP were distributed in the second and third quadrants, and all of them showed an opposite relationship with the other parameters distributed in the first and fourth quadrants (Figure 7B).

## Discussion

As an evolutionarily highly conserved molecule, melatonin has a wide range of functions in plants. Studies have revealed that melatonin plays positive roles in improving the seedling growth and development of maize (Turk and Erdal, 2015), cucumber (Bałabusta et al., 2016; Zhang R. et al., 2017), soybean (Zhang J. et al., 2017), *Malus hupehensis* Rehd. (Gong et al., 2017), watermelon (Li et al., 2017), and saffower (Amjadi et al., 2021) under adverse conditions. In addition, a high-nitrate treatment inhibited shoot height, stem diameter, shoot/root dry weight, and total dry weight, whereas the pretreatment with 0.1 mmol L^−1^ melatonin dramatically increased these parameters under high-nitrate treatment (Zhang R. et al., 2017). In the present study, the effect of melatonin on the morphological parameters in alfalfa (Figure 1) was consistent with the previous results (Zhang R. et al., 2017; Bose and Howlader, 2020). It indicates that melatonin can improve the growth and development of alfalfa under high-nitrate stress.

Ion uptake and compartmentalization are vitally important not only for improving normal growth but also for sustaining plant performance under high-salinity conditions because such adversity disturbs ion homeostasis (Adams et al., 1992). Excessive nitrogen is harmful to plants, even though nitrogen is essential for growth, development and metabolisms (Hao et al., 2015, 2017; Zhang R. et al., 2017; Wen et al., 2020). When nitrogen uptake exceeds plant assimilation capacity, plants will accumulate excessive ${\text{NO}}_{3}^{-}$-N and ${\text{NH}}_{4}^{+}$-N, which will lead to soil secondary salinization and plant salt toxicity, thus affecting growth and development (Britto and Kronzucker, 2002; Roosta and Schjoerring, 2007; Zhang R. et al., 2017). In the present study, exogenous melatonin can enhance the capacity for nitrate reduction and ammonia assimilation, thereby alleviating the damage caused by high-nitrate stress. What's more, SP was rapidly degraded by high-nitrate stress both on days 0 and 10 (Figure 3C). Studies have revealed that Pro concentration of plants increases obviously under salinity, drought, heavy metals, high temperature, and pathogen infection (Krishnan et al., 2008; Liu et al., 2020). Because it can protect cells from damage by acting as both an osmotic agent and a radical scavenger. As a multi-functional molecule, Pro accumulated during a stress episode is degraded to provide a supply of energy to drive growth once the stress is relieved (Hayat et al., 2012; Kishor and Sreenivasulu, 2014). Furthermore, exogenous Pro plays an important role in improving and enhancing plant stress resistance (Hayat et al., 2012; Hussain et al., 2021). Hence, the Pro concentration increased in the HN treatment and decreased as melatonin alleviates high-nitrate stress. The results further suggest that excessive nitrogen severely affected nitrogen concentrations and plant growth of alfalfa, and the application of melatonin reversed these changes. That is, melatonin can improve the growth of alfalfa by inhibiting and controlling the excess nitrogen under high-nitrate stress.

Phosphorus is not only a constituent of many plant substances but also provides anion equivalents and is responsible for the charge balance in plants, especially those that are salt-stressed (Roosta and Schjoerring, 2007; Zhang R. et al., 2017). The effect of melatonin on phosphorus concentration in alfalfa (Figure 2B) is consistent with the previous studies (Yuan et al., 2015; Zhang R. et al., 2017). The adverse effects of salt stress on plants include osmotic stress, ionic stress, and secondary stress (Munns and Tester, 2008; Qin and Duan, 2019). Potassium is an indispensable inorganic ion in osmotic regulation (Golldack et al., 2003). Nitrates can cause osmotic stress and potassium can mitigate such stress (Zhang R. et al., 2017). Studies have shown that calcium provides intermolecular linkages and protects cell walls and membranes (Xu et al., 2014). Melatonin also plays a vital role in protecting cell membranes (Li et al., 2012; Zhang et al., 2013; Arnao and Hernández-Ruiz, 2019; Zhao et al., 2021). What's more, melatonin increases calcium concentration and works synergistically together in plants under stress (Zhang R. et al., 2017; Vafadar et al., 2020). In the present study, the treatment with KNO_3_ and Ca(NO_3_)_2_ dramatically increased the concentrations of sodium, potassium, and calcium while melatonin limited their uptake under high-nitrate stress (Figures 2C-E). Melatonin likely regulates intracellular ion balance by controlling sodium and potassium, thus enabling alfalfa to respond to osmotic stress caused by high-nitrate stress (Zhang R. et al., 2017). In addition, calcium intake exceeds the normal requirements of alfalfa, and melatonin can suppress excessive calcium intake to a certain extent. Thus, melatonin, along with appropriate calcium, plays a positive role in protecting the cells of alfalfa.

NR, GS, GOGAT, and GDH are the key enzymes involved in nitrogen metabolism. ${\text{NO}}_{3}^{-}$-N is catalyzed to nitrite (${\text{NO}}_{3}^{-}$-N) by NR. NR is the rate-limiting enzyme in nitrogen metabolism. NR is also a substrate-inducing enzyme, which mainly occurs at the transcriptional level, and it is induced by ${\text{NO}}_{3}^{-}$-N, carbohydrates and light, etc. Plant cells quickly transfer ${\text{NO}}_{3}^{-}$-N from the cytoplasm to the chloroplasts of leaf cells or the cytoplasm of roots, where nitrite reductase (NiR) reduces ${\text{NO}}_{3}^{-}$-N to ${\text{NH}}_{4}^{+}$ (Wen et al., 2020). Then, ${\text{NH}}_{4}^{+}$ from nitrate assimilation or photorespiration is transferred to amino acids to avoid the toxic effects of ${\text{NH}}_{4}^{+}$ accumulation (Liu et al., 2013; Zhang R. et al., 2017). NiR activity in plants is very strong, generally does not accumulate in plants, but NiR is very sensitive to salt stress. And salt stress reduces the activity of NR, so nitrate ions could not be converted into nitrite ions and accumulated in plants. GS, GOGAT, and GDH play crucial roles in NH_3_ assimilation (Liu et al., 2013; Wen et al., 2020). GS catalyzes the synthesis of glutamine from ${\text{NH}}_{4}^{+}$ and glutamic acid. Furthermore, increased glutamine levels stimulate GOGAT and transfer the amides of glutamine to α-ketoglutarate, and produce glutamic acid. What's more, ${\text{NH}}_{4}^{+}$ can be assimilated via the deamination and synthesis of glutamate by GDH stimulation (Zhang R. et al., 2017; Wen et al., 2020). Although the activities of nitrogen metabolism enzymes are induced by ${\text{NO}}_{3}^{-}$-N and ${\text{NH}}_{4}^{+}$-N, the high accumulations are toxic to higher plants because excessive accumulation of ${\text{NO}}_{3}^{-}$-N and ${\text{NH}}_{4}^{+}$-N can cause strong feedback inhibition (Britto and Kronzucker, 2002; Zhang R. et al., 2017; Wen et al., 2020). Hence, high-nitrate stress inhibited the enzymes' activities, simultaneously inhibited protein synthesis and enhanced the hydrolase activities, and led to SP degradation; while melatonin significantly increased the enzymes' activities under high-nitrate stress (Figures 3B, 4). The increased activity of these enzymes is related to melatonin's enhanced antioxidant properties because GS and GOGAT proteins can be oxidatively modified (Balestrasse et al., 2006; Reiter et al., 2016; Zhang R. et al., 2017; Bose and Howlader, 2020; Zhao et al., 2021). What's more, as an endogenous metabolite that can be degraded by plants, melatonin itself can promote plant development during different stages (Turk and Erdal, 2015; Arora and Bhatla, 2017). On the other hand, melatonin directly up-regulates *NR, GS*, and *GOGAT* to change the enzymes' activities under high-nitrate stress. It further illustrates the ability of melatonin to enhance enzyme activities and promote nitrogen metabolism in alfalfa.

As a universal intracellular energy currency, ATP directly provides energy for gene expression, metabolism, transport, and signal transduction pathways in all organisms (Cao et al., 2014; De Col et al., 2017; Tripathi et al., 2018). It is widely distributed in mitochondria, chloroplasts, and cytoplasm in cells. Itis also an important signaling molecule in the communication between cells. ATP in eukaryotic cells is synthesized via photosynthesis and respiration in plants (De Col et al., 2017; Gao et al., 2019). ATP is synthesized in the mitochondria, chloroplasts, and cytoplasmic stroma of plant cells and then transferred to the extracellular matrix in a variety of ways. Plant cytolysis occurs when cells are injured, which provides a passive pathway for the release of ATP. De-phosphorylation to ADP and AMP, and rephosphorylation to ATP allow high energy fluxes based on relatively small pool sizes in the cell (Rich, 2003; De Col et al., 2017). In addition, EC modulates the metabolisms related to energy utilization and regeneration because many physiological and molecular responses in plant cells are associated with energy state (Zhu et al., 2012). Studies have demonstrated that stresses such as anoxia (Huang et al., 2005), extreme temperature (Stupnikova et al., 2006), low pH (Messerli et al., 2005), and salt (Wu et al., 2019) deregulate the physiology of the plant cell and cause ATP overconsumption. In the present study, alfalfa under high-nitrate stress consumed more ATP and produced more AMP and ADP, thus resulting in a decrease in EC. Nevertheless, melatonin can protect alfalfa from high-nitrate stress, thereby enhancing ATP regeneration systems and reducing ATP-utilizing systems. What's more, correlation analysis and principal component analysis showed that the parameters of morphology, mineral nutrition, nitrogen metabolism, and energy status were closely correlated with each other, and they were divided into two principal components to explain the mechanisms. Namely, these parameters jointly regulate the growth of alfalfa under high-nitrate stress.

**Figure 8:**
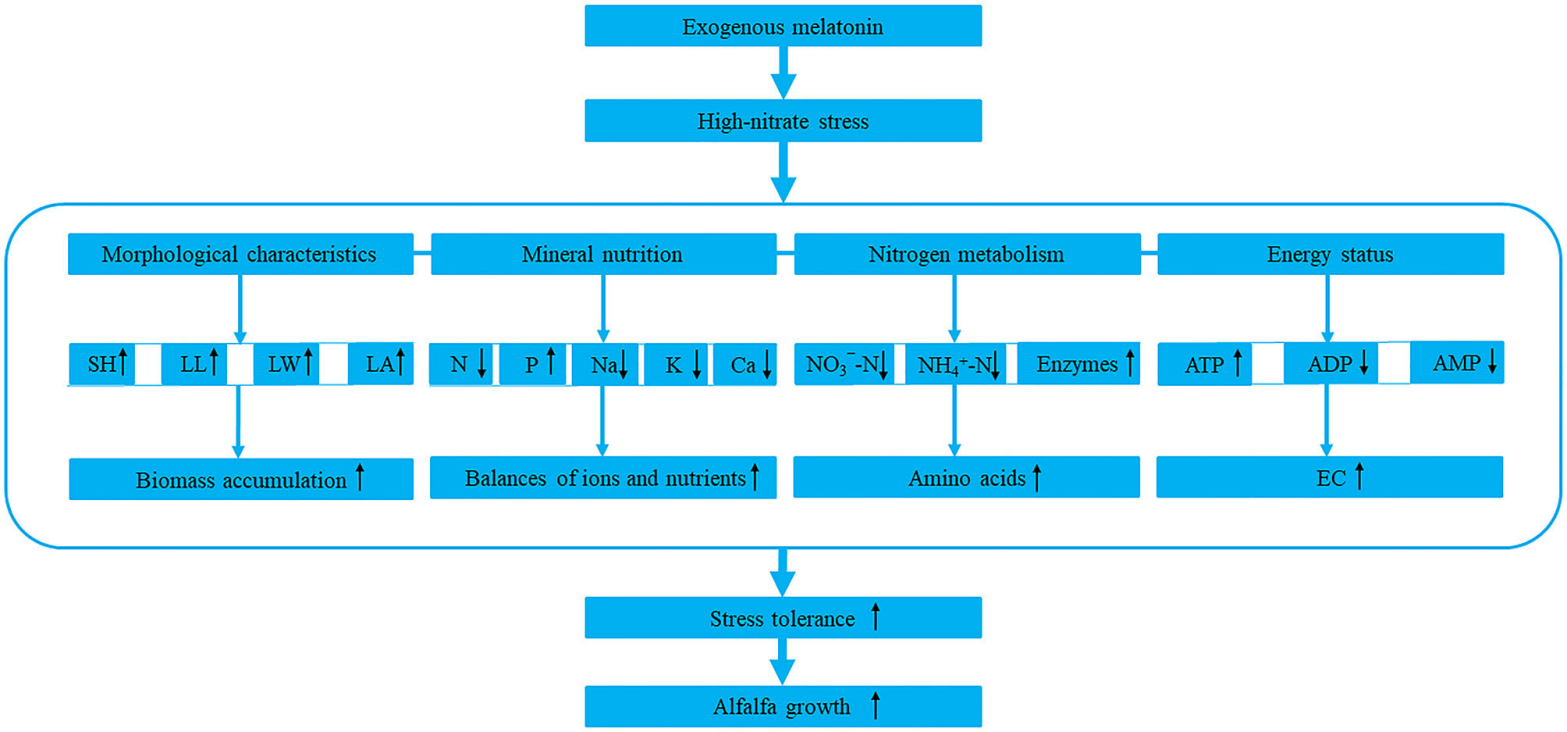
The model of melatonin response to high-nitrate stress in alfalfa. SH, LL, LW, and LA represent shoot height, leaf length, leaf width, and leaf area, respectively. N, P, Na, K, and Ca represent nitrogen, phosphorus, sodium, potassium, and calcium, respectively. ${\text{NO}}_{3}^{-}$-N and ${\text{NH}}_{4}^{+}$-N represent nitrate-nitrogen and ammonium-nitrogen, respectively. Enzymes include NR (nitrate reductase), GS (glutamine synthetase), GOGAT (glutamate synthase), and GDH (glutamate dehydrogenase). ATP, ADP, AMP, and EC represent adenosine triphosphate, adenosine diphosphate, adenosine monophosphate, and energy charge. The upward or downward black arrows represent promotion or inhibition, respectively. The blue dash indicates that the four components are closely related to each other.

Based on the above, we summarized a model of melatonin response to high-nitrate stress (Figure 8). However, as a hormone, antioxidant and signaling molecule, melatonin has complex interactions with other hormones, and they jointly regulate and control the growth and development of plant. Therefore, the specific interactions and mechanisms in alfalfa need further study.

## Conclusion

Melatonin inhibited the accumulations of ${\text{NO}}_{3}^{-}$-N and ${\text{NH}}_{4}^{+}$-N by increasing the activities of enzymes involved in nitrogen metabolism and up-regulating the expression of their related genes in alfalfa. Melatonin also modulated mineral nutrition and energy status to alleviate the damage of high-nitrate to alfalfa. Thus, melatonin improved the growth of alfalfa under high-nitrate stress. Furthermore, the parameters correlated with each other and were divided into two principal components. It indicates that melatonin has a positive effect on modulating the morphology, mineral nutrition, nitrogen metabolism, and energy status of alfalfa under high-nitrate stress.

## Data Availability Statement

The original contributions presented in the study are included in the article/supplementary material, further inquiries can be directed to the corresponding author/s.

## Author Contributions

ZC and JN conceived the ideas designed the methodology. ZC, XC, and JN conducted the experiments and analyzed the data. JN wrote the manuscript. All authors contributed to the article and approved the submitted version.

## Conflict of Interest

The authors declare that the research was conducted in the absence of any commercial or financial relationships that could be construed as a potential conflict of interest.
